# A Fundamental Ultrafast Spectroscopic Insight into Urocanic Acid Derivatives

**DOI:** 10.1021/acs.jpclett.5c00137

**Published:** 2025-02-18

**Authors:** Jack Dalton, Hans Sanders, Wybren Jan Buma, Vasilios G. Stavros

**Affiliations:** † Department of Chemistry, University of Warwick, Gibbet Hill Road, Coventry, CV4 7AL, U.K.; ‡ Van’t Hoff Institute for Molecular Sciences, University of Amsterdam, Science Park 904, 1098 XH Amsterdam, The Netherlands; § Institute for Molecules and Materials, Radboud University, Toernooiveld 7c, 6525 ED Nijmegen, The Netherlands; ∥ School of Chemistry, University of Birmingham, Birmingham, B15 2TT, U.K.

## Abstract

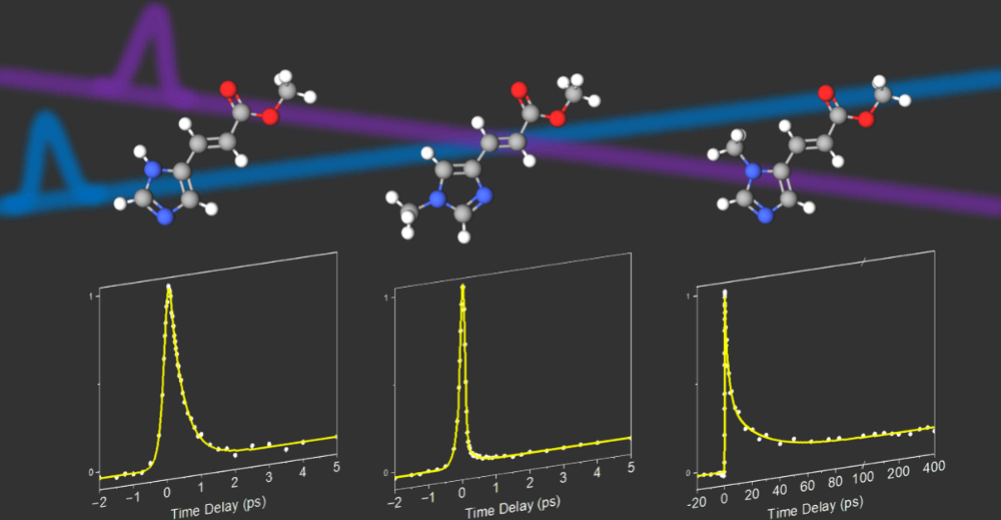

*trans*-Urocanic acid (UA) was once thought to be an ideal natural UV sunscreen filter because of its strong UV absorption and efficient nonradiative decay, in addition to its natural presence in human skin. However, its commercial use was abandoned following the discovery of the immunosuppressive properties of the *cis* isomer formed following photoexcitation. From the extensive literature accumulated over the past decades, UA serves as a perfect scaffold for developing next-generation nature-inspired UV filters by eliminating the immunosuppressive characteristics and retaining the favorable photophysics. Here, gas-phase time-resolved ion-yield and time-resolved photoelectron spectroscopy are combined to uncover the fundamental ultrafast photodynamics of three UA derivatives. We find that minor molecular adjustments between derivatives have considerable influence on the overall excited state behavior, leading to different relaxation mechanisms and lifetimes. These findings establish foundations for further molecular design and aid in the interpretation of these derivatives under complex environmental conditions.

Urocanic acid (UA) is a naturally occurring chromophore found in the outermost layer of the human epidermis in its *trans* (E) isomeric form (see Figure 1 for molecular structure).^1−3^ It has a strong UVB (ultraviolet B, 280–315 nm) absorption and is known to undergo efficient nonradiative decay via a *trans–cis* isomerization coordinate following UV irradiation (C_6_=C_7_ in Figure 1).^4−12^ These photophysical properties, coupled with the fact that UA is already present in the skin, should make UA an exemplary natural UV sunscreen. Indeed, between the 1960s and 1990s UA was incorporated in various cosmetics and commercial sunscreen formulations.^13^ However, in 1983 De Fabo and Noonan discovered that the resulting *cis* (Z) isomer is immunosuppressive, with subsequent studies corroborating this finding.^14−17^ This prompted the gradual removal of UA from cosmetic products due to growing health concerns.

**Figure 1:**
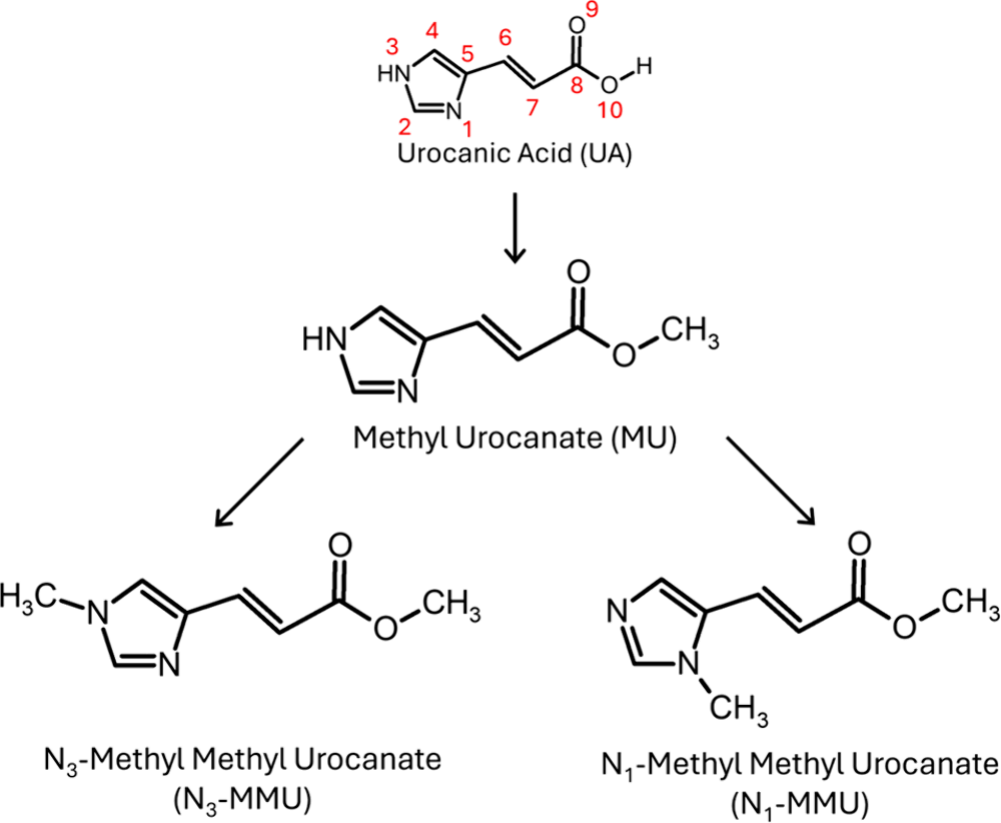
Molecular structures of urocanic acid (UA), methyl urocanate (MU), N_3_-methyl methyl urocanate (N_3_-MMU) and, N_1_-methyl methyl urocanate (N_1_-MMU).

From the understanding gleaned over the past decades, UA is well suited to be used as a scaffold for developing next generation biomimetic UV filters through judicious molecular design, removing immunosuppressive characteristics while retaining the auspicious excited state behavior. A natural first approach is to methylate the acidic proton producing methyl urocanate (MU, see Figure 1 for the structure). This substitution removes the hydrogen-bonding ability and in turn, decreases the relative stability of the photogenerated *cis* isomer.^18^ Further substitutions can involve monomethylation at either of the nitrogen atoms of the imidazole, producing N_3_-methyl methyl urocanate (N_3_-MMU, Figure 1) or N_1_-methyl methyl urocanate (N_1_-MMU, Figure 1). Recently MU, N_3_-MMU and N_1_-MMU were investigated using frequency-resolved and nanosecond time-resolved spectroscopy which proposed that their photodynamics could critically depend on two ubiquitous, close lying, ^1^nπ* and ^1^ππ* states.^18^ In particular, Fan et al. found that the imidazole substitutions drastically affect the properties of these lower lying electronic states, enabling tuning of their UV absorption properties, and hence potential applications. Furthermore, based on extracted resonance enhanced multiphoton ionization (REMPI) line widths, there appears to be photodynamics occurring on ultrafast (femtosecond) time scales that can dramatically affect the overall nonradiative decay pathways of the derivatives.

It is vital to extend our foundational *in vacuo* understanding of these UA derivatives before exploring the impact of solvent and then more complex sunscreen formulation environments in which they may be found (i.e., on cutaneous surfaces). For this reason, this work reports the results of gas-phase ultrafast (femtosecond) time-resolved ion-yield (TR-IY) and time-resolved photoelectron spectroscopy (TR-PES) to provide a unique insight into MU, N_3_-MMU and N_1_-MMU’s ultrafast photodynamics in the absence of environmental perturbation (an experimental pump–probe schematic summarizing these experiments is provided in Scheme 1). These experiments build upon the gas-phase frequency-resolved spectra and nanosecond dynamics of these molecules, uncovering the hitherto unseen ultrafast relaxation mechanisms that dictate energy dissipation following excitation to the optically bright ^1^ππ* state. Furthermore, we determine the extent of involvement of the close-lying ^1^nπ* state. This foundational basis opens the way for the interpretation of more complex environments and provides the building blocks for further molecular design.

**Scheme 1:**
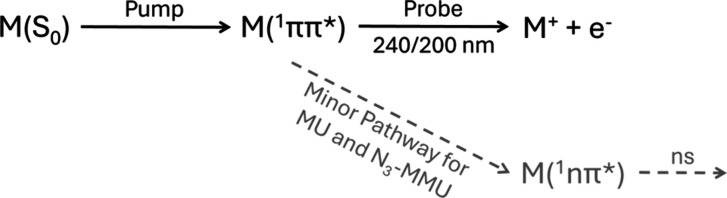
A Summary of the Pump-Probe Experiment MU, N_3_-MMU, and N_1_-MMU, denoted by ‘M’, are first photoexcited to their ^1^ππ* band origins (see text for details). The probe ionizes the photoexcited molecules to produce the parent ion, M^+^, and photoelectron, e^–^, which are detected separately.

## Methyl Urocanate

**Figure 2:**
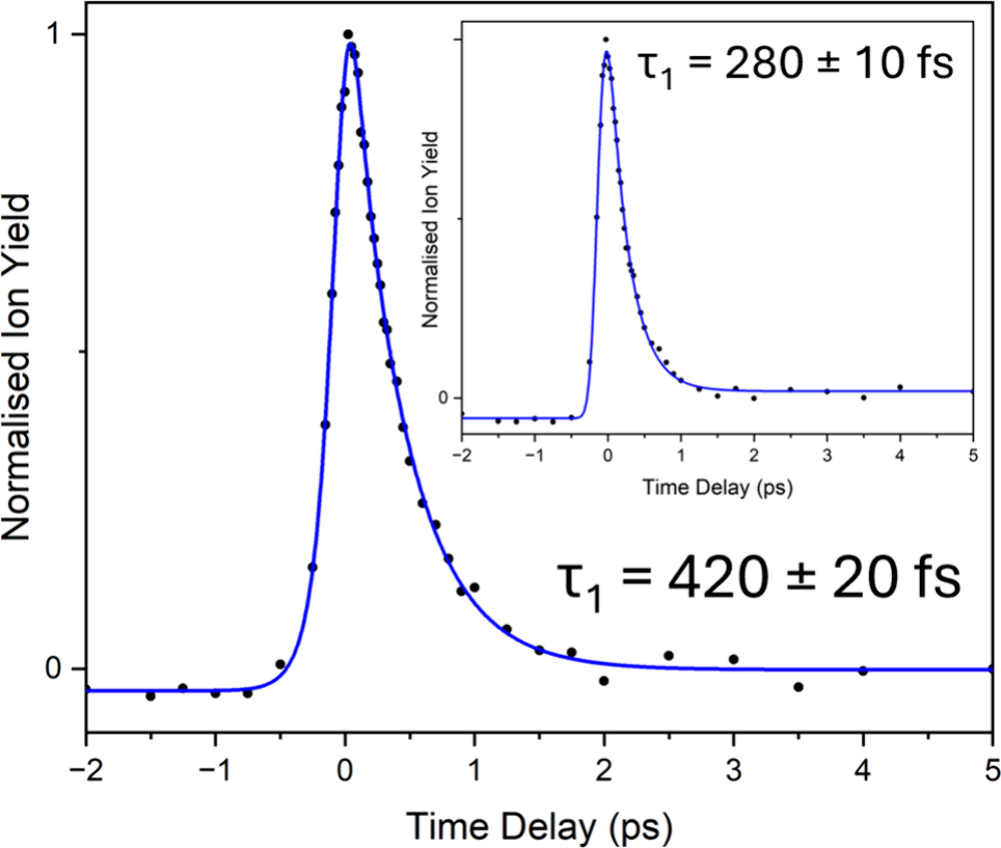
Time-resolved ion-yield (TR-IY) transient of MU photoexcited at 306 nm. The main graph is with a 200 nm probe, while the inset is with a 240 nm probe. The blue traces are fits with one exponential decay in the positive time delay and two in the negative time delay; the forward lifetime (Δ*t* > 0) is presented along with the error pertaining to one standard error between the fit and the raw data. For the 200 and 240 nm probe TR-IY transients, the greatest standard deviation is 13% for both relative to the total ion signal. The pump and probe are parallel with respect to one another and are in the plane of the detector.

In heating to 180 °C to obtain sufficient vapor pressure, MU thermally degrades resulting in additional masses recorded in the time-of-flight mass spectrum (TOF-MS, see Supporting Information, SI, Figure S1). This is similar to, but not to the same extent as UA which is known to efficiently undergo thermal decarboxylation before sufficient vapor pressure is obtained, rendering UA’s gas-phase results unreliable with this method.^19,20^ Consequently, MU was primarily investigated via TR-IY, integrating over the parent (MU^+^) ion signal. In all cases, MU was photoexcited at its band origin of 32,632 cm^–1^ using a pump pulse centered at 306 nm, populating the ^1^ππ* state, and the subsequent dynamics were tracked with a 240 or 200 nm ionizing probe.^18^Figure 2 presents the TR-IY transient with both a 200 and 240 nm probe. First, the reverse dynamics (pump–probe time delay, Δ*t*, < 0; probe excitation and 306 nm ionization) present a fast decay followed by a negative baseline offset. These reverse dynamics are observed for all TR-IY transients of all molecules studied here, which we attribute to decay involving a dissociative excited state, supported by a greater number of fragments observed in the mass spectrum at negative time delays (SI Figure S1). In the forward dynamics (Δ*t* > 0), MU displays an ultrafast decay that returns to baseline (based on subtraction of the pump and probe alone transients and addition of the background transient).

To obtain a quantitative understanding of MU’s photodynamics, the parent transients were fitted with a monoexponential decay (for the forward-going dynamics) convoluted with a Gaussian to model the instrument response (∼165 fs using a 240 nm probe and ∼200 fs using a 200 nm probe, see SI Figure S2). For Figure 2, using a 200 nm probe, this returned a lifetime of 420 ± 20 fs (with the error pertaining here, and in all future cases, to one standard error between the fit and the raw data). This lifetime is in excellent accord with the estimated lifetime of ∼400 fs obtained from the REMPI line width of 12 to 16 cm^–1^ by Fan et al. for the ^1^ππ* state.^18^ As such, and since no additional dynamics are observed here, we assign this lifetime to decay via internal conversion (IC) from the initially populated ^1^ππ* state to the ground electronic state. Fan et al. additionally observed a parent-ion transient that returned a 31 ns decay attributed to decay via a ^1^nπ* state and possibly a triplet state following the depopulation of the initially populated ^1^ππ* state.^18^ This is not observed in the present work. We suspect this is a minor decay channel, which accords with the weak parent-ion signal transient observed by Fan et al.

Interestingly, when using a 240 nm probe, an extracted lifetime of 280 ± 10 fs is obtained from the TR-IY transient (Figure 2 inset), *cf*. 420 ± 20 fs with a 200 nm probe. Through close inspection of the comparable TR-PE spectrum with a 240 nm probe (SI Figure S3), we can see that the main spectral feature is cutoff in the low energy region, suggesting that the reduction in lifetime obtained from the TR-IY transient may be attributed to the insufficient energy of the 240 nm probe to ionize from the evolving excited ^1^ππ* state. This in turn highlights a potentially steep ^1^ππ* potential energy surface (PES), driving excited state population toward the ground electronic state. We note however that this interpretation is caveated with a caution owing to the enhanced thermal degradation, resulting in the products influencing the photoelectrons observed.

## N_3_-Methyl Methyl Urocanate

**Figure 3:**
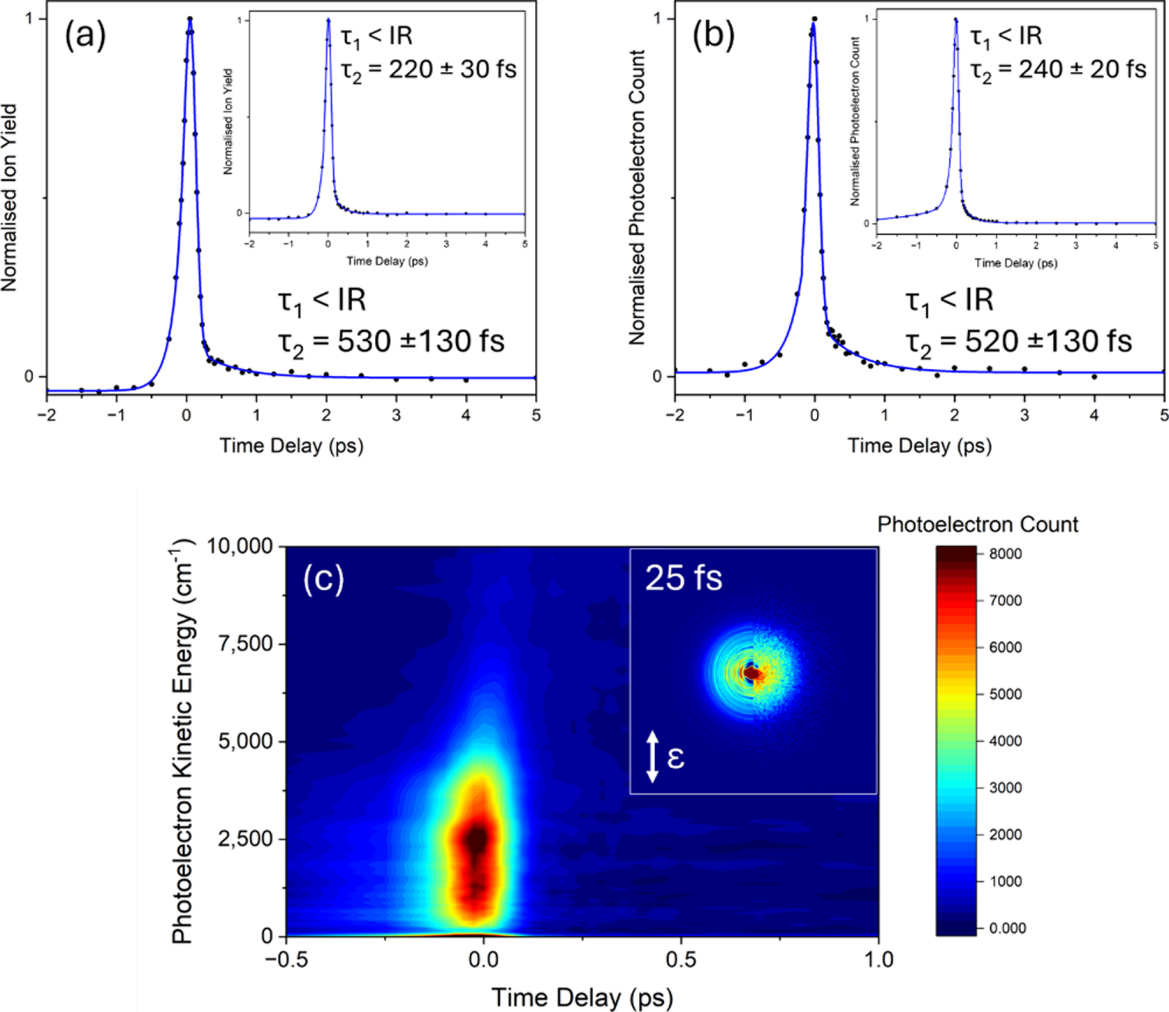
TR-IY and time-resolved photoelectron (TR-PE) transients of N_3_-MMU photoexcited at 301 nm. (a) TR-IY transient at 200 nm probe. (a) Inset: TR-IY transient at 240 nm probe. (b) TR-PE transient at 200 nm (see corresponding electron kinetic energy, eKE, false color heatmap in Supporting Information, SI, Figure S4). (b) Inset: TR-PE transient at 240 nm probe. (c) eKE false color heatmap for (b) inset showing the eKE regions contributing to the signal intensity (eKE was smoothed with a moving average of 4). (c) Inset: right half presents the recorded image, and left half presents the reconstructed slice through the original three-dimensional (3D) photoelectron distribution at Δ*t* = 25 fs (the double-headed arrow indicates the electric field polarization, ε, of the laser pulses). The blue traces in (a) and (b) are fits containing a sequential biexponential decay in the positive time delay and a parallel biexponential decay in the negative time delay; the first forward lifetime (Δ*t* > 0), τ_1_, is less than the instrument response (IR) of ∼130 fs using a 240 nm probe and ∼170 fs using a 200 nm probe (see SI Figure S2), and the second forward lifetime, τ_2_, is presented along with the error pertaining to one standard error between the fit and the raw data. For the 200 and 240 nm probe TR-IY transients, the greatest standard deviation is 13% and 9% respectively, relative to the total ion signal. The pump and probe are parallel with respect to one another and in the plane of the detector.

N_3_-MMU was photoexcited at the band origin of 32,250 cm^–1^ using a pump centered at 301 nm, populating the ^1^ππ* state.^18^ The subsequent photodynamics of the parent-ion were, as with MU, tracked with a 240 or 200 nm probe. Figure 3 (a) and (b) present the TR-IY and TR-PE transients, respectively, with both a 200 and 240 nm probe. The corresponding electron kinetic energy (eKE) false color heatmap with a 240 probe is presented in Figure 3 (c). Overall, the forward dynamics show a steep ultrafast decay followed by a weak, slightly longer, decaying feature before returning to baseline.

All transients were fit with a biexponential decay, yielding two forward lifetimes. The first, τ_1_, is near instantaneous, and within the time-resolution of our instrument (∼130 and ∼170 fs using a 240 and 200 nm probe respectively, see SI Figure S2). The second lifetime, τ_2_, is extracted as ∼500 fs when probing at 200 nm and ∼230 fs when probing at 240 nm, similarly to that seen for MU’s τ_1_. Comparing the TR-PE eKE features shown in Figure 3 (c) and SI Figure S4 with a 240 and 200 nm probe, respectively, we suggest the measured lifetime is reduced due to insufficient probe energy to ionize along the total excited state coordinate (see SI Figure S5 for additional 240 nm probe TR-PE spectra). This highlights a potentially steep ^1^ππ* PES that can drive excited state population toward the ground electronic state, akin to that seen for MU. It is also important to note the considerable reduction in intensity of the τ_2_ feature, suggesting a lower oscillator strength for ionization. A line width from the REMPI spectrum obtained by Fan et al. cannot accurately be determined as there appears to be heavy overlap in the vibronic structure, however it is reasonable to assume, given the broadness, there are ultrafast dynamics, which is confirmed here.^18^ The calculations by Fan et al. also found that there is a change in electronic excited state ordering between vertical to adiabatic energies causing the ^1^nπ* state to be lower in energy than the ^1^ππ* state, invoking the possibility of IC to the ^1^nπ*.^18^

Based on this information, the following mechanism is proposed: following excitation to the ^1^ππ*, N_3_-MMU undergoes initial movement out of the Franck–Condon region nearly instantaneously to a point on the PES whereby the ionization cross section is significantly lower. This first process accounts for τ_1_. Subsequentially, further movement along the ^1^ππ* PES occurs before undergoing IC to either the close lying ^1^nπ* state or the ground electronic state within ∼500 fs, accounting for τ_2_. Once again, Fan et al. observed a weak parent-ion transient that returned a 14 ns decay that was attributed to decay from the ^1^nπ* state or a triplet state.^18^ As with MU, this is not observed in the present work, possibly due to the inability to ionize from the ^1^nπ*. However, this 14 ns decay indicates that deactivation from the ^1^ππ* state, τ_2_, involves, at least, a minor decay channel of IC to the ^1^nπ*, possibly involving (subsequent population of) a triplet state.

## N_1_-Methyl Methyl Urocanate

**Figure 4:**
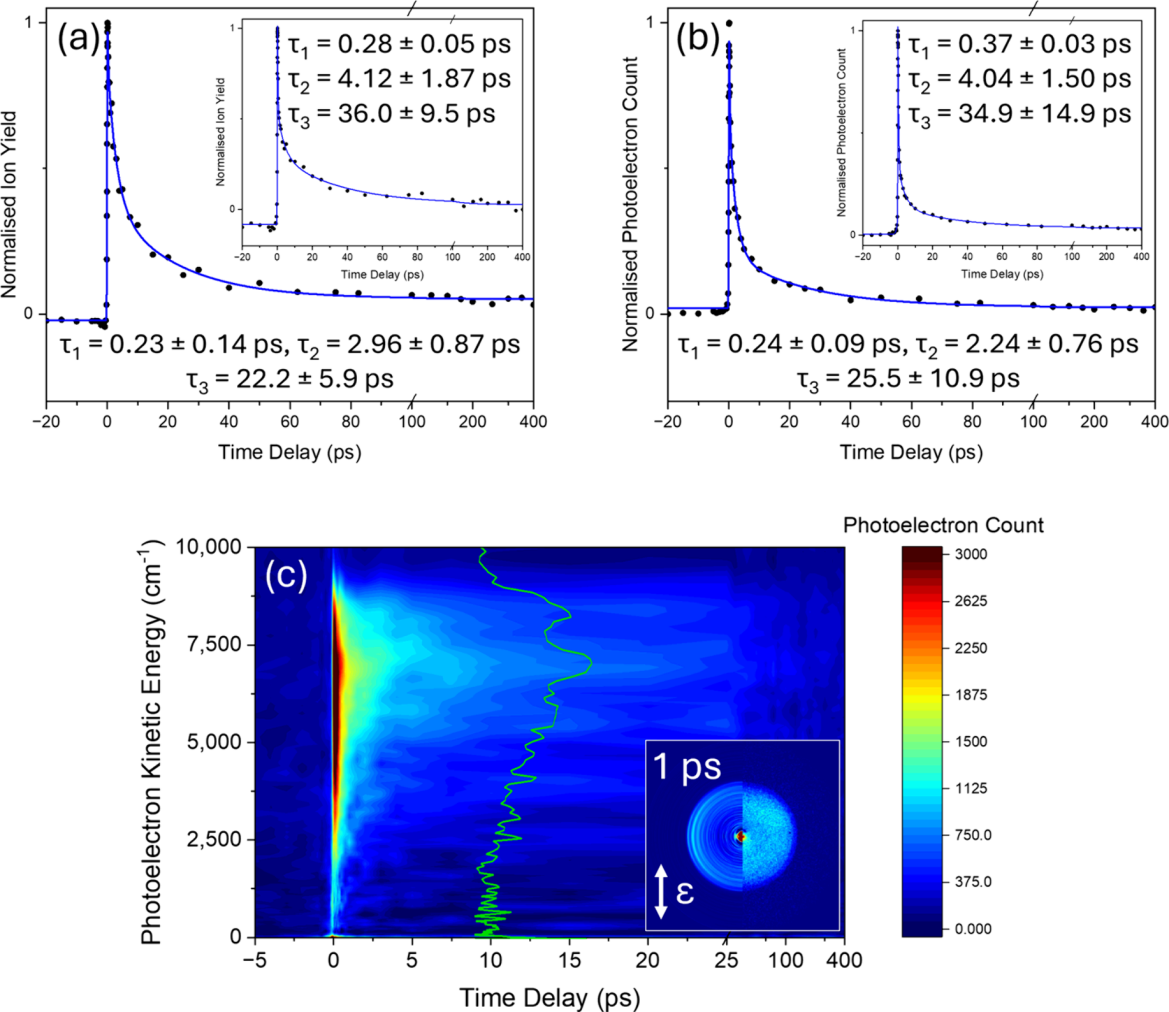
TR-IY and TR-PE results for N_1_-MMU photoexcited at 316 nm. (a) TR-IY transient at 200 nm probe. (a) Inset: TR-IY transient at 240 nm probe. (b) TR-PE transient at 200 nm (see corresponding eKE false color heatmap in SI Figure S6). (b) Inset: TR-PE transient at 240 nm probe. (c) eKE false color heatmap for (b) inset showing the eKE regions contributing to the signal intensity (eKE was smoothed with a moving average of 4). (c) Inset: right half presents the recorded image, and left half presents the reconstructed slice through the original 3D photoelectron distribution at Δ*t* = 1 ps (the double-headed arrow indicates the electric field polarization, ε, of the laser pulses). An overlay in (c) of a 10 ps TR-PE spectrum with a 240 nm probe is shown in green. The blue traces in (a) and (b) are fits containing a sequential triexponential decay in the positive time delay and a single exponential decay in the negative time delay; the forward lifetimes (Δ*t* > 0) are presented along with the error pertaining to one standard error between the fit and the raw data. For the 200 and 240 nm probe TR-IY transients, the greatest standard deviation is 29% and 7% respectively, relative to the total ion signal. (a) and (b) are linear up to 100 ps and logarithmic thereafter, while (c) is linear up to 25 ps and logarithmic thereafter. The pump and probe are parallel with respect to one another and in the plane of the detector.

A pump pulse centered at 316 nm photoexcited N_1_-MMU to its band origin, populating the ^1^ππ* state and, in parallel to MU and N_3_-MMU, a 240 or 200 nm ionizing probe monitored the subsequent excited state photodynamics.^18^Figure 4 (a) and (b) present the TR-IY transients and TR-PE transients, respectively, with both a 200 and 240 nm probe. The corresponding eKE false color heatmaps for the TR-PE transients at 200 and 240 nm probe are shown in SI Figure S6 and Figure 4 (c), respectively. The forward dynamics present a sharp drop in intensity followed by a weak, longer decay, before plateauing by ∼100 ps. There is (possibly) a persistent, but minor, positive baseline offset. At this stage, it is unclear whether, due to its low intensity, this offset is a real photodynamical feature or an error arising from an incomplete background subtraction.

All N_1_-MMU transients were fitted with a triexponential decay fitting model producing three forward lifetimes of: τ_1_ ≈ 0.3 ps, τ_2_ ≈ 3 ps and τ_3_ ≈ 30 ps. The REMPI spectrum of N_1_-MMU by Fan et al. displayed well-resolved bands with line widths of ∼2 cm^–1^ for the ^1^ππ* state with two close lying conformers contributing to the overall REMPI signal.^18^ The line widths correspond to an estimated lower bound for the^1^ππ* state lifetime of ∼3 ps, of which both the τ_2_ and τ_3_ lifetimes found here fall within. In contrast to both MU and N_3_-MMU, the calculations by Fan et al. found no reordering of electronic excited states, leaving the ^1^nπ* state higher in energy than the ^1^ππ* state, implying relaxation does not occur via the ^1^nπ* state.

The TR-PE heatmaps in Figure 4 (c) and SI Figure S6, and selected TR-PE spectra presented in SI Figure S7 provide valuable insight into the energy changes occurring within N_1_-MMU as it relaxes. Within the first picosecond there is a minor change in energy that can be seen on the high eKE edge of the spectra, indicating an initial mild change in potential energy. Furthermore, the TR-PE data in Figure 4 and SI Figure S7 display possible vibronic structure that is present throughout all time delays and can also be clearly seen in the reconstructed slice in Figure 4 (c) inset and the derived TR-PE spectrum (Figure 4, Green). Importantly, since this vibronic structure is retained throughout the excited state dynamical processes of N_1_-MMU, it implies minimal structural changes and that there is no change in electronic character, thus indicating that all excited state dynamics are occurring from the initially populated ^1^ππ* state.

From these data, it is possible to narrow down the possible relaxation mechanism that occurs in N_1_-MMU following photoexcitation to the ^1^ππ* state. One possibility is that τ_1_ and τ_2_ relate to different movements along the ^1^ππ* PES that have different rates; τ_1_ is likely initial movement out of the Franck–Condon region and τ_2_ is further intramolecular vibrational redistribution (IVR) along the ^1^ππ* PES before arriving at a conical intersection (CI) with the ground electronic state. τ_3_ is thus IC through the ^1^ππ*/S_0_ CI, repopulating the electronic ground state. Alternatively, the two different conformers observed may have distinctly different ^1^ππ* state lifetimes such that the τ_2_ and τ_3_ observed here each relate to one of the two conformers, and τ_1_ being a convolution of evolution out of the Franck–Condon region and IVR from both conformers. Both of these possibilities align with the lack of nanosecond photodynamics previously observed which indicate that no further dynamical processes occur.^18^

To conclude, ultrafast time-resolved ion-yield and time-resolved photoelectron spectroscopy proved critical in determining the photodynamics of methyl urocanate (MU), N_3_-methyl methyl urocanate (N_3_-MMU) and, N_1_-methyl methyl urocanate (N_1_-MMU) following photoexcitation to their ^1^ππ* state at the band origin. The previously unknown ultrafast relaxation mechanisms, including the extent of involvement from the elusive and close lying ^1^nπ* state was unveiled. A summary of the lifetimes for the observed photodynamic processes is presented in Table 1. MU and N_3_-MMU were found to primarily undergo internal conversion (IC) back to the ground electronic state within ∼420 and ∼500 fs, respectively, possibly via a steep ^1^ππ* potential energy surface. In both cases, there is also a minor decay channel involving IC to the nearly ^1^nπ* state that either lives on the nanosecond time scale or decays to a nearby triplet state that decays on a nanosecond time scale. N_1_-MMU bypasses the ^1^nπ* state entirely, repopulating the electronic ground state within ∼30 ps via IC from the initially populated ^1^ππ* state.

**Table 1:** A Summary of the TR-IY Lifetimes Using a 200 nm Probe for MU, N_3_-MMU, and N_1_-MMU

	τ_1_/fs	τ_2_/ps	τ_3_/ps
MU	420 ± 20	-	-
N_3_-MMU	<170 (IR)	0.53 ± 0.13	-
N_1_-MMU	230 ± 140	2.96 ± 0.87	22.2 ± 5.9

The results presented here prove crucial for developing a foundational understanding of how minor initial molecular design changes to urocanic acid drastically affect the resulting excited state photodynamics. Substitution of the acidic proton and further substitution of the N_3_ imidazole nitrogen results in ultrafast relaxation from the initially populated ^1^ππ* state and potential population of the ^1^nπ* state. As such, further modifications to these positions could likely retain the favorably fast relaxation from the ^1^ππ* state while aiming to avoid population of the unfavorably long-lived ^1^nπ* state. Modification to the N_1_ imidazole nitrogen appears to result in a lack of the ^1^nπ* state population at the cost of slower deactivation from the initially populated ^1^ππ* state. This knowledge provides a basis for more extensive molecular functionalization that could offer an improved ability to suppress the immunosuppressive properties while maintaining favorable photophysics. Some examples of future modifications include using bulkier substituents in the current methyl substituted areas to possibly reduce immunosuppressive properties by reducing the probability of binding and activating receptors, or adding substitutions to the carbons on the acrylic double bond that have shown to improve the rate of photoisomerisation for similar molecules.^21−23^ Finally, uncovering the quantum yields of different relaxation pathways would be of great value and interest and thus merits further investigation.

## Experimental Methods

The experimental setup has been described in detail elsewhere, so only details specific to this experiment are presented here.^23,24^ A Ti:sapphire oscillator (Spectra-Physics Tsunami) and regenerative amplifier system (Spitfire XP) produce 3 mJ, ∼40 fs pulses at a rate of 1 kHz and centered at 800 nm. The 3 W output is split into three 1 W beams, two of which are used here. The first 1 W beam pumps an optical parametric amplifier (Light Conversion, TOPAS-C) to generate the pump pulse, centered at 306, 301, or 316 nm to photoexcite methyl urocanate (MU), N_3_-methyl methyl urocanate (N_3_-MMU) or, N_1_-methyl methyl urocanate (N_1_-MMU), respectively, at their band origins. The second 1 W beam either pumped another TOPAS-C to generate a 240 nm probe or was successively frequency converted by using a series of type I, type II, and type I β-barium borate crystals to produce a 200 nm probe. The pump pulse is temporally varied with respect to the probe pulse via a hollow UV-enhanced aluminum retroreflector mounted on a motorized delay stage (Physik Instrumente), allowing for a maximum pump–probe time delay (Δ*t*) of 1.2 ns. Laser powers were set to ensure single photon dynamics. The polarizations of the pump and probe were parallel with respect to each other and in the plane of the detector. With the pump and probe polarizations set to magic angle (54.7°) with respect to each other, the observed dynamics are unchanged, indicating the absence of rotational artifacts (SI Figure S8).

The pump and probe intersect a molecular beam produced from seeding MU, N_3_-MMU, or N_1_-MMU heated to 180 °C, 150 °C, or 130 °C respectively, into 1.5 bar helium (see reference for molecule synthesis).^18^ This gas mixture was expanded into vacuum with an Even-Lavie solenoid valve and was subsequently passed through a 2 mm diameter skimmer.^25,26^

For time-resolved ion-yield (TR-IY) measurements, cations are accelerated from the point of pump–probe intersection toward a metal anode detector (Del Mar Photonics MCP-MA25/2) coupled with a microchannel plate (MCP). A digital oscilloscope (LeCroy LT372 Waverunner) measured the detector’s output, and signal relating to the parent ion (MU^+^, N_3_-MMU^+^, or N_1_-MMU^+^) was recorded as a function of Δ*t* to produce a TR-IY transient.

For time-resolved photoelectron (TR-PE) measurements, a velocity map imaging (VMI) setup based on a design by Eppink and Parker was used.^27^ Here, photoelectrons are accelerated and focused onto a position-sensitive detector consisting of two MCPs coupled to a phosphor screen (Photek VID-240) and imaged by a CCD camera (Basler A-312f). With this design, electrons with the same initial kinetic energy have the same radial position. The two-dimensional images produced were reconstructed to their original three-dimensional distribution via a polar onion peeling algorithm, before deriving a one-dimensional photoelectron spectrum.^28^ The detector was calibrated from scaling xenon’s photoelectron spectrum to coincide with the energy of its well-known ionization states.^29^ One-dimensional photoelectron spectra, which consider all orientations, were integrated as a function of Δ*t* to produce a TR-PE transient.

Dynamical information from the TR-IY and TR-PE transients were extracted using an exponential decay model convoluted with a Gaussian instrument response (IR).^23^ The IR of the experiment was estimated from the cross-correlation of the pump and probe with ammonia (see SI Figure S2). The IR was found to be between ∼125 fs to ∼200 fs, dependent on the pump and probe wavelengths.

## Supporting Information Available

Low resolution mass-spectra; additional TR-PE spectra; magic angle TR-IY transients; cross-correlation data (PDF)
