# Supplementary material for: A Fundamental Ultrafast Spectroscopic Insight into Urocanic Acid Derivatives
Source: J Phys Chem Lett. 2025 Feb 18;16(8):2016–22. doi: 10.1021/acs.jpclett.5c00137 (PMC11873962; doi:10.1021/acs.jpclett.5c00137)
Supplement: Supplementary file 1 — jz5c00137_si_001.pdf [file jz5c00137_si_001.pdf]

Supporting Information for: A Fundamental Ultrafast Spectroscopic Insight into Urocanic Acid Derivatives

Jack Dalton[1], Hans Sanders[2], Wybren Jan Buma[2,3], Vasilios G. Stavros[4*]

[1]Department of Chemistry, University of Warwick, Gibbet Hill Road, Coventry, CV4 7AL, U.K.

[2]Van't Hoff Institute for Molecular Sciences, University of Amsterdam, Science Park 904, 1098 XH Amsterdam, The Netherlands.

[3]Institute for Molecules and Materials, Radboud University, Toernooiveld 7c, 6525 ED Nijmegen, The Netherland

[4]School of Chemistry, University of Birmingham, Birmingham, B15 2TT, U.K.

*Correspondence: v.stavros@bham.ac.uk (V.G.S.).

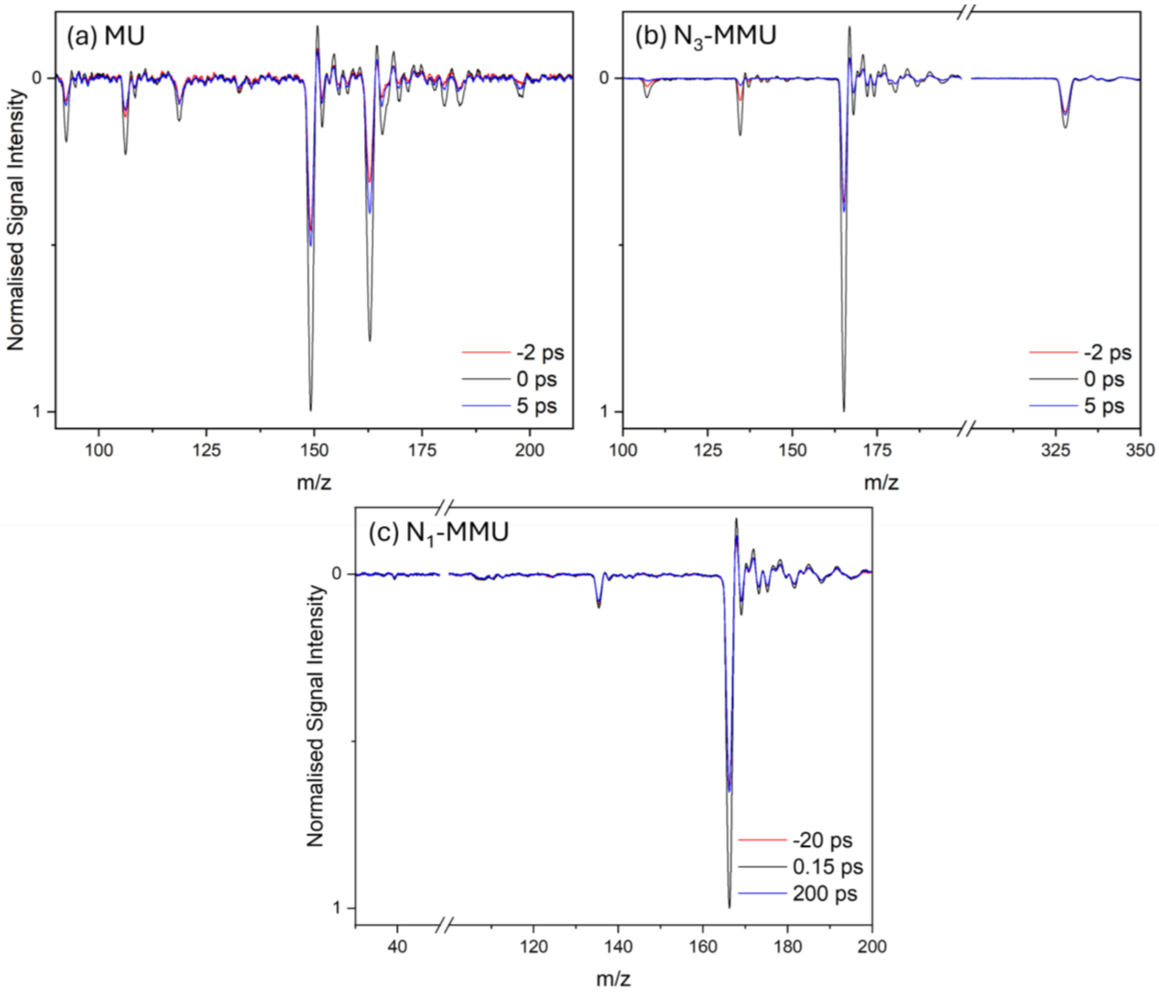

Figure S1. Low resolution mass spectra of (a) methyl urocanate (MU), (b) N_3-methyl methyl urocanate (N_3-MMU), and (c) N_1-methyl methyl urocanate (N_1-MMU) pumped at 306 nm, 301 nm, and 316 nm, respectively, and probed at 240 nm. Three pump-probe time delays were chosen to show the mass spectrum at the baseline before pump-probe dynamics (red line), with maximum pump-probe dynamics (black line) and the baseline after pump-probe dynamics (blue line). For MU in (a), the parent ion (MU^+) is the largest peak at ~150 m/z and the second largest peak is a result of thermal decomposition. The major thermal decomposition product gradually grows in amplitude throughout a day of being heated while the parent signal simultaneously drops in amplitude. The major thermal decomposition product also presented different pump-probe dynamics to the parent. For N_3-MMU in (b), a dimer of two parent molecules is seen at ~330 m/z. In some cases, the fragment peaks are greater at the baseline before pump-probe dynamics than at the baseline after pump-probe dynamics, highlighting the potential fragmentation caused by the 240 nm probe that results in the negative offset seen in the reverse ($\Delta t < 0$) dynamics of the time-resolved ion-yield (TR-IY) transients.

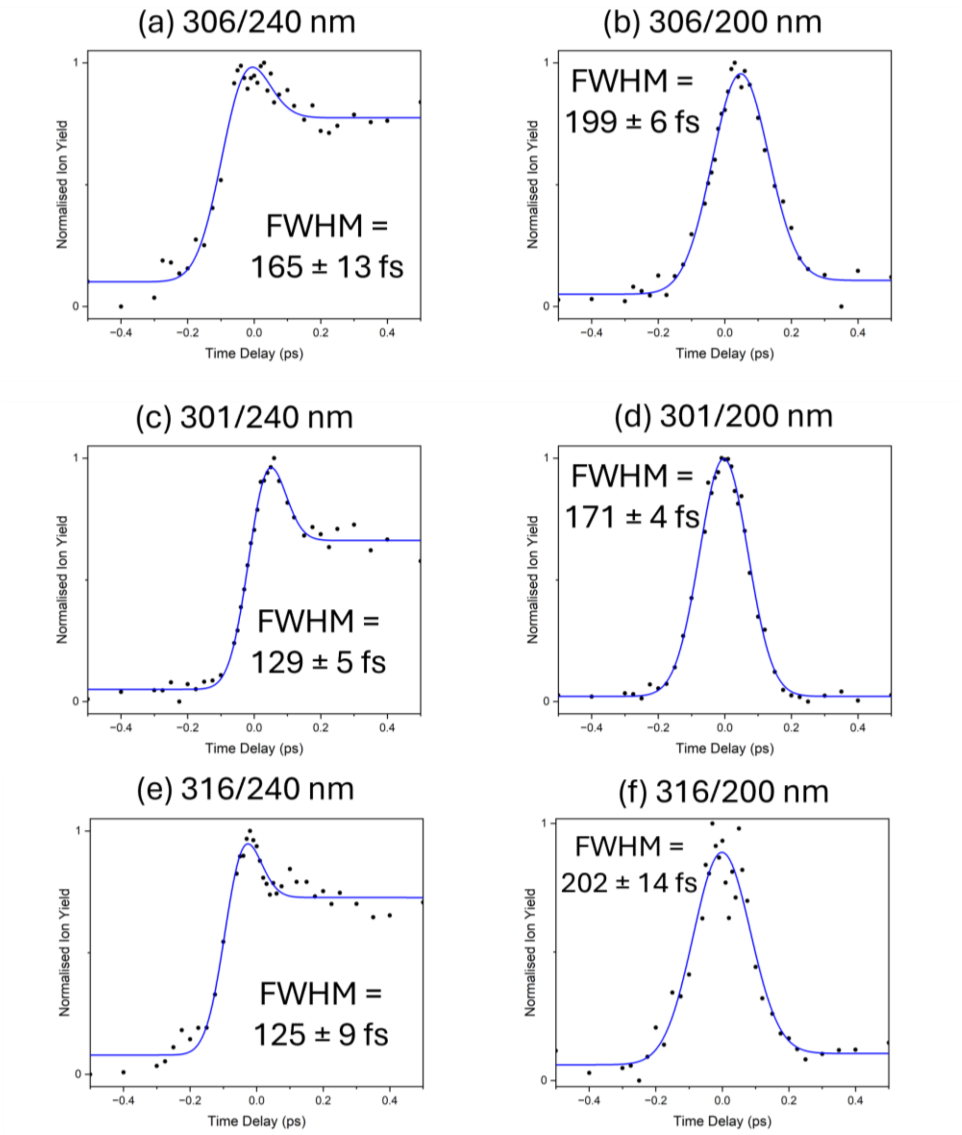

Figure S2. TR-IY cross-correlation with ammonia to estimate the temporal resolution of the experiment. Here, 5% ammonia/helium gas mixture is expanded into vacuum *via* the Even-Lavie solenoid valve and subsequentially excited and ionised with varying pump-probe time delays. The pump and probe wavelengths are shown above each graph (pump/probe). The baseline offset is likely due to population of a long-lived Rydberg state with the pump.[1] The data is fitted with a Gaussian summed with a single (positive going) long-lived exponential decay (using the same model for other exponential decays fits) to account for the long-lived state population. Shown for each graph is the full width half maximum (FWHM) of the fitted Gaussian and the presented errors pertain to one standard error between the fit and the raw data. The polarisation of the pump and probe are parallel with respect to each other and in the plane of the detector.

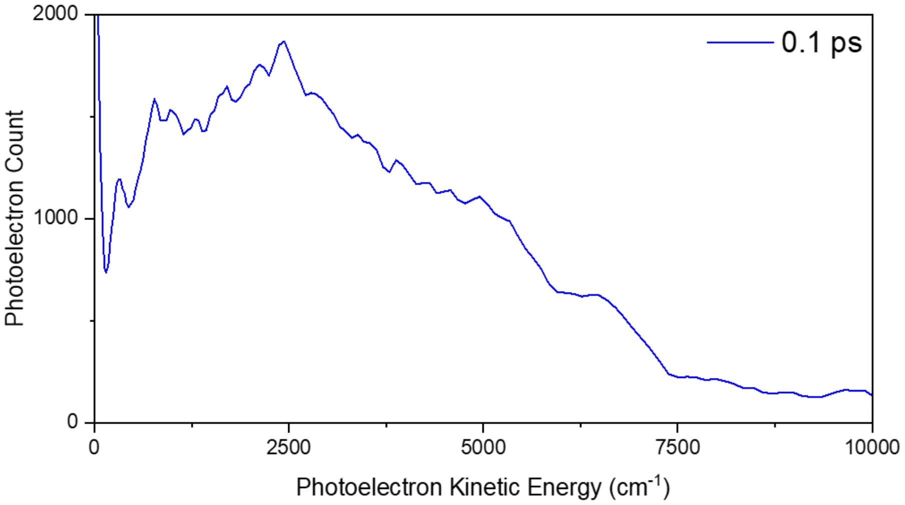

Figure S3. Time-resolved photoelectron (TR-PE) spectrum of MU photoexcited at 306 nm and probed at 250 nm at a Δ*t* = 0.1 ps. Due to the large number of fragments, clusters and thermal decomposition products (Fig. S1), this data is only presented to support the observation that using a 240 nm probe does not provide sufficient energy to ionise all population on the excited state potential energy surface. The pump and probe are parallel with respect to one another and in the plane of the detector. Electron kinetic energy (eKE) was smoothed with a moving average of 4.

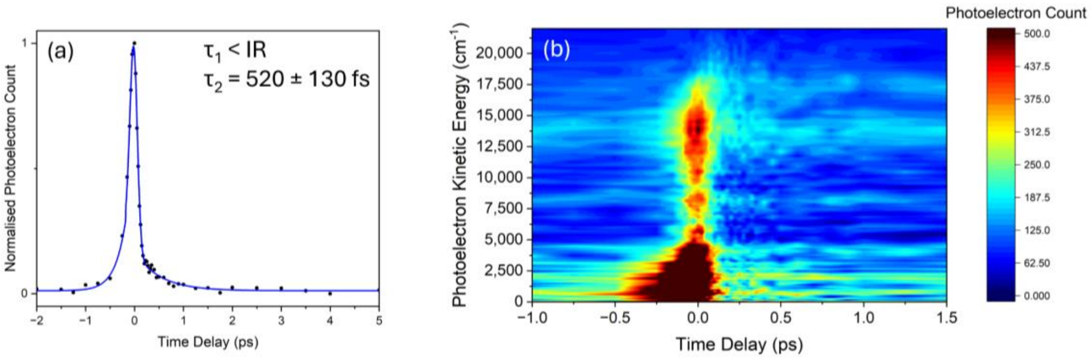

Figure S4. TR-PE transient, (a), integrated over the corresponding eKE false colour heatmap, (b), of N_3-MMU photoexcited at 301 nm and probed at 200 nm. The blue trace is a fit consisting of a sequential biexponential decay in the positive time delay and a parallel biexponential decay in the negative time delay; the first forward lifetime (Δ*t* > 0), τ_1, is less than the instrument response (IR) of ~170 fs (see SI Fig. S2), and the second forward lifetime, τ_2, is presented along with the error pertaining to one standard error between the fit and the raw data. eKE was smoothed with a moving average of 4. The pump and probe are parallel with respect to one another and in the plane of the detector.

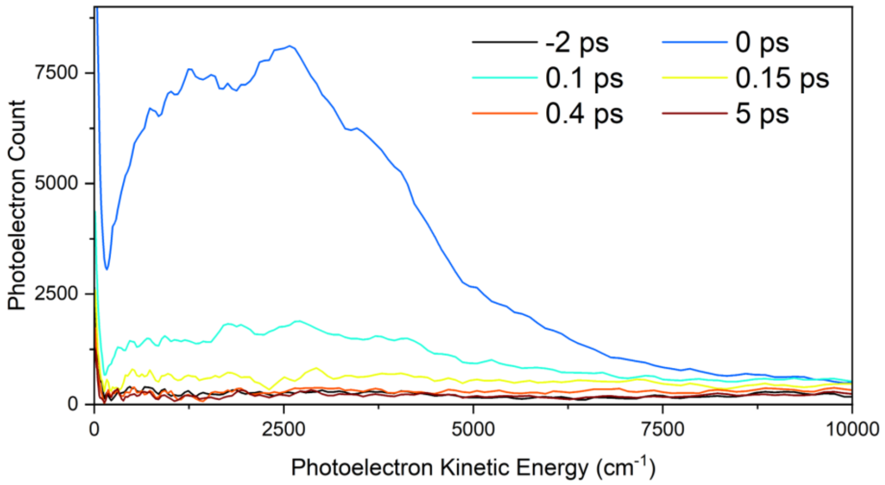

Figure S5. TR-PE spectra of N_3-MMU photoexcited at 301 nm and probed at 240 nm at the following Δ*t*: -2, 0, 0.1, 0.15, 0.4 and 5 ps. eKE was smoothed with a moving average of 4. The pump and probe are parallel with respect to one another and in the plane of the detector.

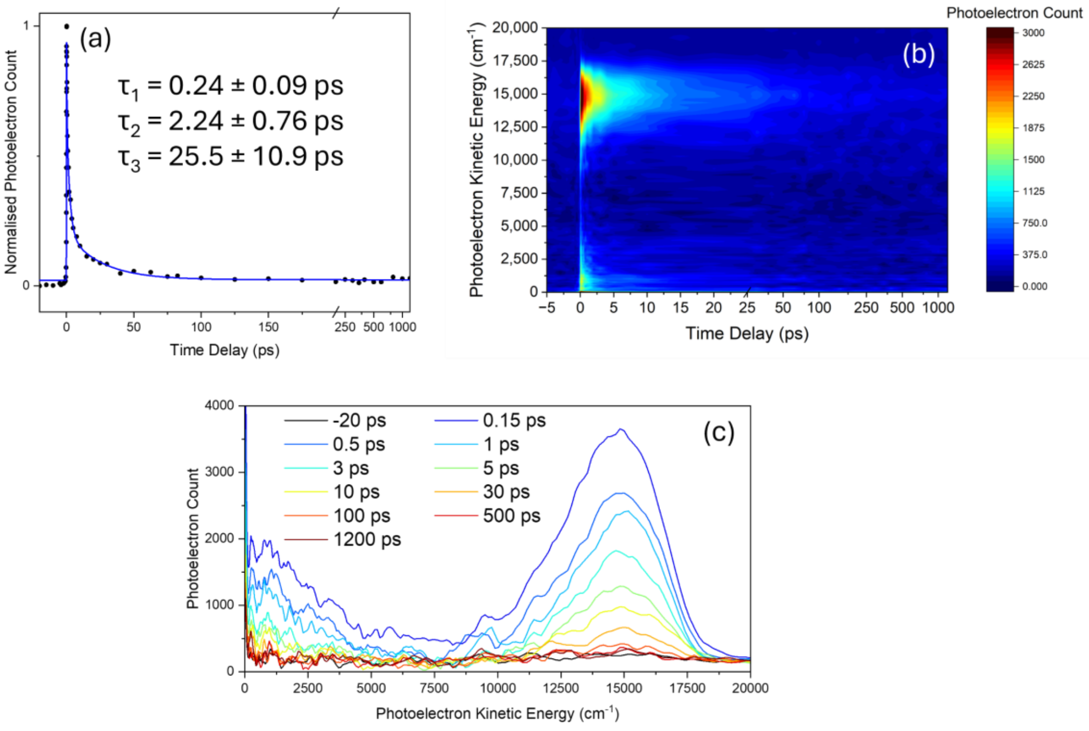

Figure S6. TR-PE results for N_1-MMU photoexcited at 316 nm and probed at 200 nm. (a) TR-PE transient fitted with a sequential triexponential decay in the positive time delay and a parallel biexponential decay in the negative time delay; the forward lifetimes (Δ*t* > 0) are presented along with the error pertaining to the fitting. (b) The corresponding eKE false colour heatmap for (a) showing the eKE regions contributing to the signal intensity (eKE was smoothed with a moving average of 4). (c) TR-PE spectra at selected pump-probe time delays. The pump and probe are parallel with respect to one another and in the plane of the detector. The TR-PE transient presented here is from an integral over all eKE, although transients integrated over only the low eKE or higher eKE feature return the same fitting lifetimes. This suggests that the low eKE feature is a result of ionisation to a higher-lying ionisation state.

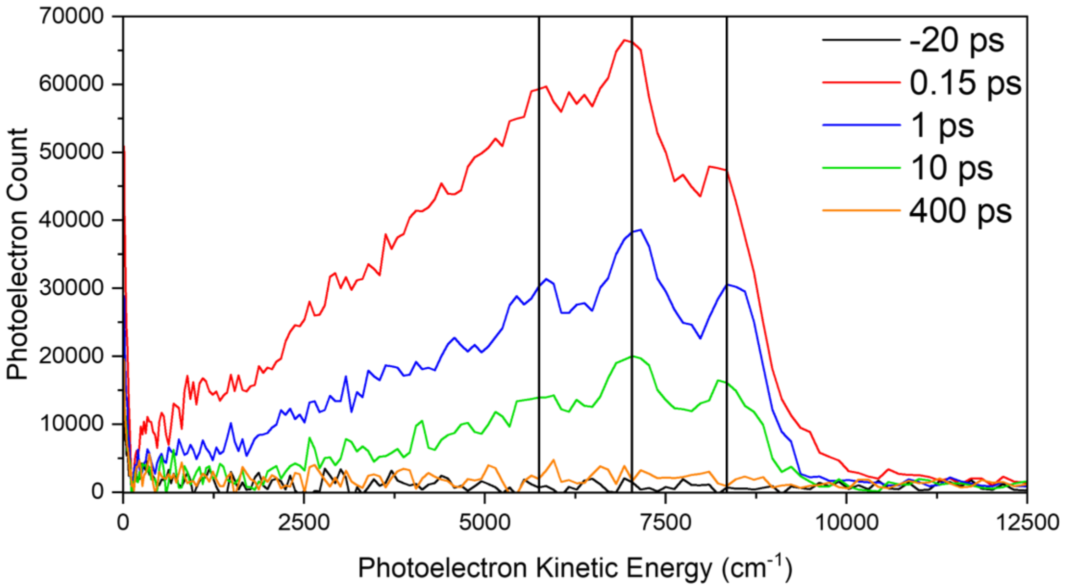

Figure S7. TR-PE spectra of N_1-MMU photoexcited at 316 nm and probed at 240 nm at the following Δ*t*: -20, 0.15, 1, 10 and 400 ps. The vibrational structure with spacing of ~1,300 cm^{-1} is highlighted with three vertical black lines. eKE was smoothed with a moving average of 4. The pump and probe are parallel with respect to one another and in the plane of the detector. From these well-resolved spectra, an approximation of N_1-MMU's ionisation potential (IP) can be obtained from $E_{pump} + E_{probe} - eKE_{max} = IP$, where E_{pump} and E_{probe} is the energy of the pump and probe, respectively, and eKE_{max} is the maximum eKE obtained from the spectra. The IP for N_1-MMU is determined to be ~63,300 cm^{-1}.

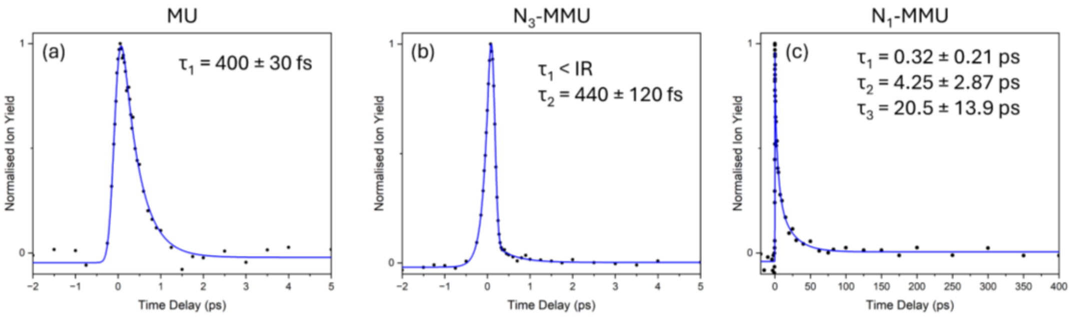

Figure S8. TR-IY transients of: (a) MU photoexcited at 306 nm, (b) N_3-MMU photoexcited at 301 nm, and (c) N_1-MMU photoexcited at 316 nm. In all cases, the probe was 200 nm. The pump and probe are at magic angle (54.7°) with respect to one another and the pump is in the plane of the detector. The blue trace is a fit with one for (a), two for (b) or three for (c) sequential exponential decays in the positive time delay and two in the negative time delay; the forward lifetime (Δ*t* > 0) is presented along with the error pertaining to the fitting. τ_1 for N_3-MMU is less than the instrument response (IR) of ~170 fs. The same dynamics are seen (within error) at parallel pump and probe, indicating the absence of rotational artifacts.

References

(1) Dobber, M. R.; Buma, W. J.; de Lange, C. A. Two-Color Picosecond Time-Resolved (2 + 1') Resonance-Enhanced Multiphoton Ionization Photoelectron Spectroscopy on the B1E'' and C' 1A1' States of Ammonia. *J. Phys. Chem.* **1995**, *99*, 1671–1685.
